# Mobile Plasmid Mediated Transition From Colistin-Sensitive to Resistant Phenotype in *Klebsiella pneumoniae*

**DOI:** 10.3389/fmicb.2021.619369

**Published:** 2021-02-15

**Authors:** Baoyue Zhang, Bing Yu, Wei Zhou, Yue Wang, Ziyong Sun, Xiaojun Wu, Shiyun Chen, Ming Ni, Yangbo Hu

**Affiliations:** ^1^CAS Key Laboratory of Special Pathogens and Biosafety, Center for Biosafety Mega-Science, Wuhan Institute of Virology, Chinese Academy of Sciences, Wuhan, China; ^2^University of Chinese Academy of Sciences, Beijing, China; ^3^Department of Pathogen Biology, School of Basic Medicine, Tongji Medical College, Huazhong University of Science and Technology, Wuhan, China; ^4^Department of Laboratory Medicine, Tongji Hospital, Tongji Medical College, Huazhong University of Science and Technology, Wuhan, China; ^5^Department of Respiratory and Critical Medicine, Renmin Hospital of Wuhan University, Wuhan, China; ^6^Department of Infectious Diseases, Tongji Hospital, Tongji Medical College, Huazhong University of Science and Technology, Wuhan, China

**Keywords:** multidrug resistance, CRKP, mgrB, colistin, phenotype transition

## Abstract

Multidrug-resistant bacteria, including carbapenem-resistant *Klebsiella pneumoniae* (CRKP), are becoming an increasing health crisis worldwide. For CRKP, colistin is regarded as “the last treatment option.” In this study, we isolated a clinical CRKP strain named as *K. pneumoniae* R10-341. Phenotyping analysis showed that this strain could transit from a colistin-sensitive to a resistant phenotype by inserting an IS*4* family IS*Kpn72* element into the colistin-resistance associated *mgrB* gene. To investigate the mechanism of this transition, we performed genome sequencing analysis of the colistin-sensitive parental strain and found that 12 copies of IS*Kpn72* containing direct repeats (DR) are located on the chromosome and 1 copy without DR is located on a multidrug-resistant plasmid pR10-341_2. Both types of IS*Kpn72* could be inserted into the *mgrB* gene to cause colistin-resistance, though the plasmid-derived IS*Kpn72* without DR was in higher efficiency. Importantly, we demonstrated that colistin-sensitive *K. pneumoniae* strain transferred with the IS*Kpn72* element also obtained the ability to switch from colistin-sensitive to colistin-resistant phenotype. Furthermore, we confirmed that the IS*Kpn72*-containing pR10-341_2 plasmid was able to conjugate, suggesting that the ability of causing colistin-resistant transition is transferable through common conjugation. Our results point to new challenges for both colistin-resistance detection and CRKP treatment.

## Introduction

Multidrug-resistant pathogenic bacteria, such as carbapenem-resistant *Klebsiella pneumoniae* (CRKP), are increasingly becoming a health crisis worldwide (Nordmann et al., 2011; Pitout et al., 2015). Polymyxins, including polymyxin B and polymyxin E (colistin), have been regarded as “the last treatment option” for CRKP (Biswas et al., 2012). Polymyxins are lipopeptide antibiotics targeting the lipopolysaccharide (LPS) of the bacterial outer membrane, the main component of the Gram-negative bacterial cell wall (Hancock, 1997; Li et al., 2006).

The increase in infections by polymyxin-resistant bacteria has become a great challenge to clinical treatment (Antoniadou et al., 2007; Bogdanovich et al., 2011; Macesic et al., 2020). In *K. pneumoniae*, the most common polymyxin-resistance mechanism is achieved by LPS modification, which decreases the negative charge of LPS and reduces its affinity to polymyxins (Velkov et al., 2014; Liu et al., 2017). The mobile colistin resistance gene (*mcr-1*) is the first reported plasmid-mediated colistin resistance gene (Liu et al., 2016), which encodes a phosphoethanolamine-lipid A transferase catalyzing the addition of phosphoethanolamine (PEtN) to lipid A (Liu et al., 2016, 2017). To date, 10 variants (*mcr*1-10) have been identified on a wide variety of transferable plasmids (Wang et al., 2018, 2020; Yang et al., 2018; Zhong et al., 2018; Lei et al., 2020), leading to the widespread diffusion of *mcr*-mediated colistin-resistance (Zhong et al., 2018; Nang et al., 2019). Chromosome-mediated colistin-resistance has also been characterized by the involvement of a small transmembrane protein MgrB and the two-component systems (TCSs) PhoPQ, PmrAB, and CrrAB (Gunn and Miller, 1996; Olaitan et al., 2014). To the best of our knowledge, in contrast to *mcr* genes, chromosome-mediated colistin-resistance mechanisms are considered to be stable and have not been reported to be transferred or mobile to other bacteria. However, inactivation of the *mgrB* gene has been widely identified from clinical colistin-resistant clinical *K. pneumoniae* strains (Cannatelli et al., 2013; Gaibani et al., 2014; Olaitan et al., 2014).

In this study, we isolated a multidrug resistant *K. pneumoniae* strain named R10-341 with high frequency (∼10^–6^) of colistin heteroresistance (El-Halfawy and Valvano, 2015; Halaby et al., 2016). Genetic and molecular analyses identified that insertion of an IS*Kpn72* element into the *mgrB* gene was responsible for the acquisition of colistin resistance. We further analyzed IS*Kpn72* copies in this strain and demonstrated that the IS*Kpn72* element is derived from a mobile plasmid and suggested that this mobile plasmid has the ability to render transition from colistin-sensitive to resistant phenotype in *K. pneumoniae*.

## Materials and Methods

### Bacterial Strains, Plasmids, and Primers

The *K. pneumoniae* strains named R10-341 and 7097 used in this study were isolated from the sputum samples collected from the Tongji Hospital, Hubei Province, China. The *K. pneumoniae* strains were grown in LB medium with 100 μg/mL ampicillin at 37°C.

For plasmid constructions, the p15A *ori* from plasmid pACYC184 (Rose, 1988) and the streptomycin-resistance gene from pTargetF (Jiang et al., 2015) were PCR amplified, respectively. These two fragments were assembled as the linearized vector p15A-Sm by overlap PCR, which was then cloned with IS*pla* (IS fragment from pR10-341_2) or IS*chr* (IS fragment from chromosome) fragment using the ClonExpress II One Step Cloning Kit (Vazyme) to generate two plasmids named p15A-Sm-IS*pla* and p15A-Sm-IS*chr* respectively. All primers used in this study are listed in Supplementary Table 1.

### Sequence Typing and Colistin Resistant Gene Detection

Multilocus sequence typing (MLST) for the *K. pneumoniae* R10-341 strain was performed as described (Diancourt et al., 2005).^1^ Colistin resistance associated genes were detected by PCR. Each of the *mcr* genes was amplified using two pairs of primers. Genes encoding the two-component systems were amplified by PCR and confirmed by DNA sequencing in comparison with the *K. pneumoniae* HS11286 strain (Accession: NC_016845). All primers are listed in Supplementary Table 1.

### Drug Susceptibility Test

MICs of antibiotics (except colistin) for *K. pneumoniae* R10-341 were determined using the broth microdilution method according to the Clinical and Laboratory Standards Institute (CLSI) guidelines (CLSI document M100-S28)^2^. The susceptibility to colistin was tested according to the guidelines of European Committee on Antimicrobial Susceptibility Testing (EUCAST)^3^. Briefly, 100 μL Cation-adjusted Mueller-Hinton Broth containing 2-fold diluted antibiotics was added to a 96-well plate, followed by the addition of 100 μL bacterial cells (10^5^∼10^6^CFU/mL) to each well. The 96-well plate was incubated at 37°C for 16–24 h. The lowest concentration of antibiotic with complete inhibition (clear broth) was regarded as the MIC.

### Genome DNA Extraction, Whole Genome Sequencing, and Bioinformatics Analysis

The *K. pneumoniae* R10-341 strain was first spread onto a LB plate. A single colony was selected and cultured in LB medium at 37°C. Genomic DNA was extracted using a bacterial genomic DNA extraction Kit (Tiangen). Genome DNA sequencing was performed by both Hiseq X Ten (Illumina) and MinION (Oxford Nanopore Technologies) platforms according to a standard protocol provided by Illumina and Oxford Nanopore Technologies. The off-machine data of Nanopore sequencing is converted to fastq format through the Albacore software in the MinKNOW software package^4^ (Payne et al., 2019). After filtering to obtain clean reads, these reads are randomly selected and aligned with the Nucleotide Sequence Database. *De novo* genome assembly was performed with Unicycler v0.4.7 (Wick et al., 2017). NCBI Prokaryotic Genomes Annotation Pipeline (PGAP) was used to annotate assembled genome sequence and to identify genes related to conjugation (Tatusova et al., 2016). Antibiotic resistance genes and plasmid replicons were respectively identified by ResFinder v3.0 (Zankari et al., 2012) and PlasmidFinder v2.0 (Carattoli et al., 2014) from the Center for Genomic Epidemiology website^5^. Sequence reads for the whole-genome sequencing are available from the NCBI Sequence Read Archive (PRJNA655367).

### IS Element Analysis

For analyzing the IS elements in *K. pneumoniae* R10-341, the IS element inserted into the *mgrB* gene in colistin resistant colonies was first identified by PCR and DNA sequencing. This IS element sequence was analyzed in ISfinder database^6^ and was named as IS*Kpn72* based on suggestion from ISfinder. The sequence of the ISKpn72 element was then aligned with the *K. pneumoniae* R10-341 genome sequence using BLASTn.

Amino acid sequences of transposases used in the phylogenetic tree analyses of the IS elements were downloaded from ISfinder database. Phylogenetic tree was constructed based on average distance using Jalview 2.11 (Waterhouse et al., 2009).

### Conjugation Analysis

Since *K. pneumoniae* R10-341 is resistant to several antibiotics but is relatively sensitive to tetracycline, we constructed a tetracycline-resistant (TcR) *E. coli* K-12 strain as the recipient in conjugation experiment using a CRISPR/Cas9 system (Jiang et al., 2015). Briefly, the *E. coli* K-12 strain was first transformed with a Cas9 expressing plasmid pCas, and the sgRNA expressing plasmid pTargetF-EclacZ (targeting the *lacZ* gene) was then co-transformed with a DNA repairing fragment containing tetracycline-resistance gene from pACYC184 plasmid (Rose, 1988). The colonies resistant to tetracycline were screened. A single colony from 50 μg/ml tetracycline-containing LB agar plate was confirmed by DNA sequencing and was named as K12-TcR for subsequent conjugation tests.

Conjugation was performed by mixing an overnight donor (*K. pneumoniae* R10-341) and logarithmic phase recipient (K12-TcR) at a ratio of 4:1 in a total volume of 1 mL as described (Wu et al., 2019). The mixture was then concentrated and spotted onto LB agar without antibiotics at 37°C for 2–4 h to allow conjugation to occur. Since the pR10-341_2 plasmid contains streptomycin resistance gene (Table 1), the conjugated bacterial mixture was plated on LB agar containing 50 μg/ml tetracycline and 50 μg/ml streptomycin to screen for transconjugants carrying plasmid pR10-341_2. Transconjugants were confirmed by PCR using primers paired to *K. pneumoniae* R10-341 (Kpn-F-FR), K12-TcR (Ec-F-FR), and pR10-341_2 plasmid (P-F-FR), respectively.

**TABLE 1:** MIC values of different antibiotics to *K. pneumoniae* R10-341.

Antibiotic name	Antibiotic class	MIC (μg/ml)	Resistance gene	Gene location
Ampicillin	Beta lactams	>256	*bla*SHV-11 *bla*KPC-2 *bla*CTX-M-27	Chromosome pR10-341_2
Kanamycin	Aminoglycoside	128	–	–
Streptomycin	Aminoglycoside	256	*aadA*	pR10-341_2
Gentamicin	Aminoglycoside	>128	–	–
Rifampin	Rifamycin	>256	*arr*-2	pR10-341_2
Chloramphenicol	Chloramphenicol	128	*cat*3	Chromosome
Ciprofloxacin	Quinolones	>256	*qnrB*	pR10-341_2
Trimethoprim	Sulfonamides	>256	*dfrA12*	pR10-341_2
Tetracycline	Tetracyclines	16	–	–
Erythromycin	Macrolides	>256	–	–

## Results

### Characterization of a Multi-Drug Resistant *Klebsiella pneumoniae* R10-341 Strain

**FIGURE 1:**
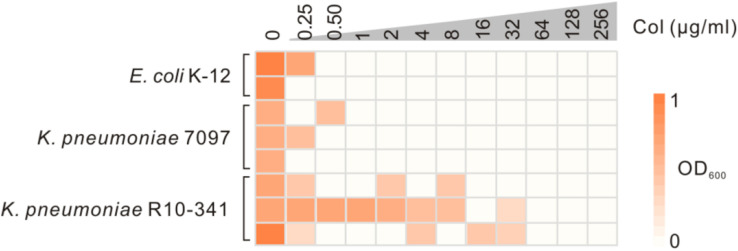
Sensitivity test of the *K. pneumoniae* R10-341 strain to colistin. *E. coli* K-12 and *K. pneumoniae* 7097 were used as control strains. The depth of the color increases with the OD_600_ values.

The *K. pneumoniae* R10-341 strain was a clinical isolate from a sputum sample collected before antibiotic treatment from Tongji Hospital in Wuhan, China. This *K. pneumoniae* R10-341 strain was classified as ST11 and was resistant to different classes of antibiotics, including beta lactams, aminoglycosides, chloramphenicol, rifamycin, quinolones, sulfonamides and macrolides (summarized in Table 1). When testing the minimal inhibitory concentration (MIC) of colistin for this strain, we observed that some of the wells in the 96-well plate tested were resistant, while other wells showed colistin-sensitive phenotype (Figure 1). To exclude the possibility that the tested strain was a mixture of colistin sensitive and resistant, DNA sequences of known colistin resistance associated genes were tested in *K. pneumoniae* R10-341 parental strain. As summarized in Table 2, *phoPQ*, *pmrAB*, *crrAB*, and *mgrB* genes were the same as those in drug-sensitive strain, and the *mcr1-8* genes could not be successfully amplified in *K. pneumoniae* R10-341 strain. To further exclude the possibility of bacterial contamination, we streaked the R10-341 strain onto LB plate and selected different single colonies. Similar results were obtained for all these single colonies (data not shown), which suggested that the appearance of colistin resistant colonies for *K. pneumoniae* R10-341 strain was due to a colistin heteroresistance (CHR).

**TABLE 2:** Genes related to colistin resistance in *K. pneumoniae* R10-341.

Resistance mechanism	Gene	Gene functions	Detection results
Lipid A assembly PEtN	*mcr-1*	Phosphatidylethanolamine-lipid A transferase	No product
	*mcr-2*		No product
	*mcr-3*		No product
	*mcr-4*		No product
	*mcr-5*		No product
	*mcr-6*		No product
	*mcr-7*		No product
	*mcr-8*		No product
L-Ara4N and PEtN synthesis and modified LPS pathway	*mgrB*	PhoPQ negative-regulate protein	WT*
	*phoPQ*	TCS (Two-Component System)	WT*
	*pmrAB*	TCS	WT*
	*crrAB*	TCS	WT*

*WT indicates gene had the 100% DNA sequence identity in coding region and a ∼150 bp promoter region as these in the reference strain K. pneumoniae HS11286.

### Insertion of an IS*4* Family Transposon Element Into *mgrB* Gene Generated Colistin-Resistant Colonies

**FIGURE 2:**
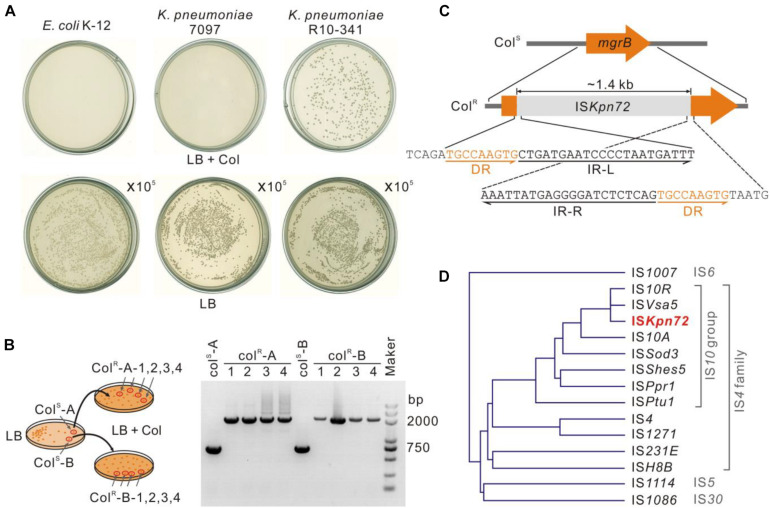
Insertion of an IS*Kpn72* transposon element into the *mgrB* gene in colistin-resistant strain**. (A)** Colonies of *E. coli* K-12, *K. pneumoniae* 7097 and *K. pneumoniae* R10-341 streaked on LB plates containing 100 μg/mL colistin. Each plate was streaked with 100 μL of logarithmic phase bacteria. As a control, 100 μL of 10^5^ diluted bacteria was also spread onto LB plates without colistin. **(B)** Confirmation of acquisition of colistin resistance for the *K. pneumoniae* R10-341 strain. Two separated colistin-sensitive colonies were spread onto two LB plates containing 100 μg/mL colistin. Fragment insertions of the *mgrB* gene in four colonies from each plate were tested by PCR. **(C)** Insertion of the IS*Kpn72* element into the *mgrB* gene in *K. pneumoniae* R10-341 colistin-resistant colonies. **(D)** Phylogenetic tree of the IS elements based on amino acid sequences of transposases.

To further characterize the *K. pneumoniae* R10-341 strain, we tested the growth of this strain on LB plates containing 100 μg/mL colistin. Consistent with MIC testing, some colonies (∼10^–6^) grew on the plate containing colistin, but no colonies were obtained from *E. coli* K-12 nor from another clinical isolate named *K. pneumoniae* 7097 on the colistin-containing plate (Figure 2A). To test the mechanism of this colistin resistance, we selected two colonies of the *K. pneumoniae* R10-341 strain from LB plate without colistin and then streaked onto LB plates containing 100 μg/mL colistin. Again, some colonies from both strains can grow on LB plates containing colistin. We then isolated four colonies from each of these two colistin-containing plates and sequenced the *phoPQ*, *pmrAB*, and *mgrB* genes (Figure 2B). Surprisingly, the amplified *mgrB* fragments from colistin-resistant colonies were all ∼1.4 kb longer than that from parental colistin-sensitive strains (Figures 2B,C).

Next, we sequenced this ∼1.4 kb inserted fragment. Sequence alignment in the NCBI database showed this fragment encodes an IS*4* family transposase. Further analysis of this ∼1.4 kb inserted fragment in ISfinder suggested that this insertion sequence could be named as IS*Kpn72* and classified into the IS*10* group in the IS*4* family, as its sequence is >95% identical to IS*10R* (Figure 2D). In accordance with this analysis, we identified 22 bp inverted repeats (IR) at both the left and right ends of this insertion fragment and 9 bp direct repeats (DR) around the insertion site (Figure 2C).

### *K. pneumoniae* R10-341 Carries the IS*Kpn72* Element Both in the Chromosome and Plasmid DNA

The R10-341 strain can become colistin-resistant by inserting the IS*Kpn72* element into the *mgrB* gene, but we do not know the source of the IS*Kpn72* element. We therefore sequenced the genome of the original colistin-sensitive *K. pneumoniae* R10-341 strain. We obtained a 5.3 Mb chromosome DNA and two plasmid sequences (named pR10-341_1 and pR10-341_2, respectively), which are 5.3Mb, 10.06 kb, and 236.3 kb with G + C contents of 57.46, 55.07, and 52.72%, respectively. According to the PlasmidFinder database, pR10-341_1 and pR10-341_2 harbored ColRNAI and IncR replicon sequence, respectively. Several antibiotic resistance genes were identified both on the chromosome and plasmids, which were in consistent with our drug resistance tests (Table 1). Genome sequencing analysis confirmed that colistin-resistance related genes, including *mgrB*, *phoPQ*, and *pmrAB*, were all the same as the *K. pneumoniae* reference strain HS11286 (Genome accession: NC_016845), which further supports our hypothesis that the colistin-sensitive strain acquired resistance in the presence of colistin.

**FIGURE 3:**
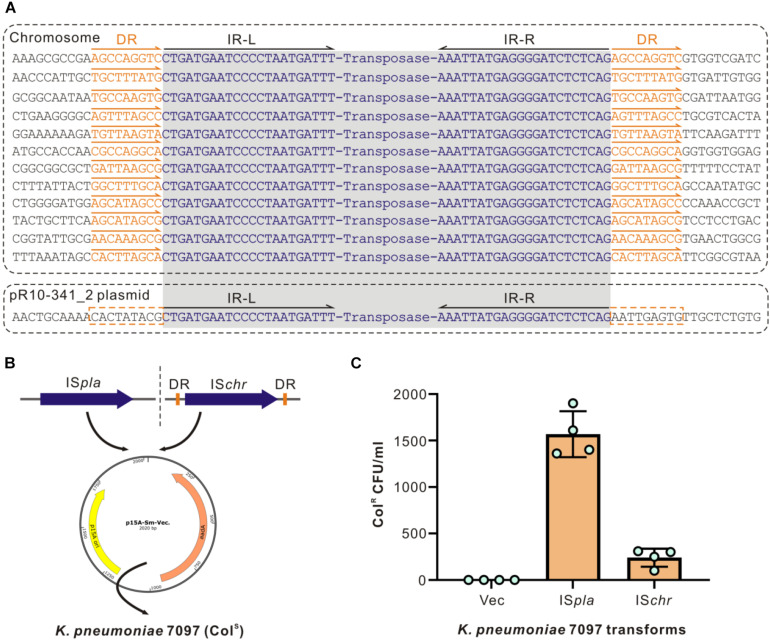
Different types of ISs have different insertion efficiencies. **(A)** Schematic diagram of IS*Kpn72* elements located on chromosome and P2 plasmid. IR sequences are shown in blue and the DR sequences are indicated in orange. **(B)** Schematic diagram for cloning two forms of the IS*Kpn72* element into colistin-sensitive *K. pneumoniae* 7097 strain. **(C)** Acquired colistin-resistance for *K. pneumoniae* 7097 strain transformed with different IS*Kpn72* elements. Overnight grown bacteria were spread onto plates containing 100 μg/mL colistin, and bacterial colony forming unit (CFU) numbers were calculated. Representative data from two-independent experiments with four technique replicates are shown.

In searching for the IS*Kpn72* element sequence in the whole genome we obtained, we found 12 copies of this IS*Kpn72* element on the chromosome, and 1 copy on the plasmid pR10-341_2. Similar to that observed in the colistin-resistant R10-341 strain, all copies of the IS*Kpn72* element contain a pair of 22 bp-length imperfect terminal inverted repeats (IR) (Figure 3A). Surrounding the 12 copies of the IS*Kpn72* element located on the chromosome are 9-bp direct repeated (DR) sequences. In contrast, the plasmid encoding the IS*Kpn72* element only contains IR sequences but not the 9 bp-DR sequences (Figure 3A). Therefore, we assumed that the copy without DR on pR10-341_2 might be the root of all the IS*Kpn72* copies on the chromosome. These analyses suggested that the IS*Kpn72* element had already been inserted into the chromosome in the parental colistin-sensitive strain.

### Both the DR-Containing and DR-Missing IS*Kpn72* Elements Can Be Inserted Into the *mgrB* Gene

Whether these existing IS*Kpn72* elements could be inserted into the *mgrB* gene to cause colistin-resistance is the next question. We transformed plasmids cloned with either the IS*Kpn72* element from pR10-341_2 or an IS*Kpn72* element from the *K. pneumoniae* R10-341 chromosome (named as IS*pla* and IS*chr*, respectively) into the colistin-sensitive strain *K. pneumoniae* 7097 (Figure 3B). In contrast to the parental *K. pneumoniae* 7097 strain, the transformation of the plasmid containing either IS*pla* or IS*chr* into this colistin-sensitive strain resulted in the growth of some colistin-resistant colonies. Colony forming units (CFU) on LB agar containing 100 μg/mL colistin revealed that the plasmid-derived IS*Kpn72* was more efficient than the chromosome-derived one in inserting into the *mgrB* gene to acquire colistin-resistance (Figure 3C). These data suggest that both IS*pla* and IS*chr* can be inserted into *mgrB* gene to acquire the colistin-resistance phenotype and IS*pla* without DRs had a higher efficiency.

### The Resistance-Acquiring Mechanism May Be Potently Disseminated Among Bacteria

**FIGURE 4:**
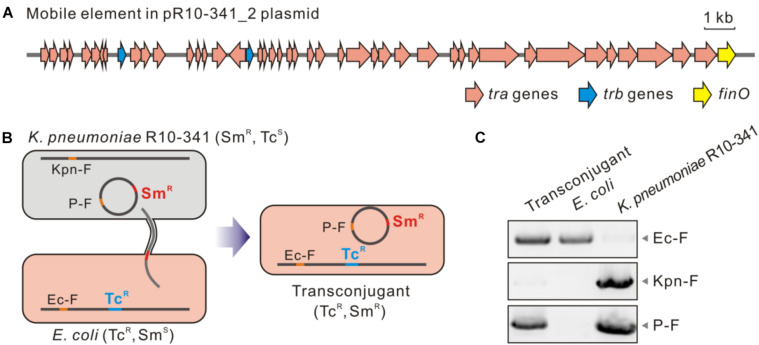
Transconjugation of the pR10-341_2 plasmid from *K. pneumoniae* R10-341. **(A)** Genes involved in mobility of the pR10-341_2 plasmid. **(B)** Strategy used in conjugation transfer analysis. **(C)** PCR confirmation of transconjugant. Data of amplified products for Ec-F, Kpn-F, and P2-F are shown.

The next question is whether the IS*Kpn72* element on the pR10-341_2 plasmid could be horizontally transferred to acquire the colistin-resistant phenotype. Sequence alignment of the pR10-341_2 plasmid from the NCBI database showed high similarity to the conjugative multidrug resistant plasmid pR46-270 in a *K. pneumoniae* R46 isolate (GenBank: CP035776.1). Accordingly, a conjugative system consisting of *tra*, *trb*, and *finO* genes were encoded by the plasmid (Figure 4A). To confirm the transferability of the pR10-341_2, we used *K. pneumoniae* R10-341 as the donor and a tetracycline-resistant *E. coli* K12-TcR strain as the recipient to verify plasmid conjugation (Figure 4B). As expected, the *E. coli* K12-TcR strain containing the pR10-341_2 plasmid was successfully obtained (Figure 4C), suggesting the pR10-341_2 plasmid was transferable. Together, these results demonstrated that the *K. pneumoniae* R10-341 was able to disseminate the ability to switch from colistin-sensitive to resistant phenotype by transferring an IS containing plasmid.

## Discussion

Colistin-resistant bacteria are becoming an increasing threat to healthcare especially in hospitals (Antoniadou et al., 2007; Bogdanovich et al., 2011; Zhong et al., 2018; Nang et al., 2019). Previous studies have focused on mechanisms of drug-resistance and how to detect these resistant strains (Olaitan et al., 2014; Poirel et al., 2017). In this study, we identified a colistin-sensitive *K. pneumoniae* R10-341 strain with high frequency (∼10^–6^) of colistin heteroresistance. The *K. pneumoniae* R10-341 strain was classified as ST11, which is the most widely prevalent CRKP genotype in China and contains the *bla*KPC-2 gene, which encodes the KPC family carbapenem-hydrolyzing class A beta-lactamase (Qi et al., 2011; Liu et al., 2018). We showed that this *K. pneumoniae* R10-341 strain could transit from a colistin-sensitive to a resistant phenotype by the insertion of an IS*Kpn72* element into the *mgrB* gene. Importantly, we characterized this IS*Kpn72* element in a mobile plasmid, which showed high similarity to the conjugative plasmid pR46-270 (Wu et al., 2019). These analyses suggest that the ability of transiting from colistin-sensitive to resistant may be disseminated through plasmid transfer.

Heteroresistance has been reported to lead to failures in antibiotic treatment (Band et al., 2016; Band et al., 2019). In CRKP, colistin heteroresistance could be achieved by inserted inactivation and deletion or point mutations in *mgrB*, *phoP*, *phoQ*, *lpxM*, and *yciM* genes (Jayol et al., 2015; Halaby et al., 2016). Similar as previous studies, we found that the colistin heteroresistance in *K. pneumoniae* R10-341 was also caused by inactivation of chromosomal encoded *mgrB* gene, but we characterized that this inactivation was mediated by a mobile plasmid derived IS element. Therefore, our result is the first to report that the colistin heteroresistance is able to be transferred or spread via plasmid mobilization.

Colistin-resistance in *K. pneumoniae* can be achieved by chromosome- or plasmid-encoded genes (Ah et al., 2014; Olaitan et al., 2014; Rebelo et al., 2018). Chromosome-encoded mutations are considered to be stable (Gunn et al., 1998), while plasmid encoded *mcr* genes can be potentially transferred across bacterial species to cause direct colistin-resistance (Liu et al., 2016; Zhong et al., 2018). Being different from spreading of drug-resistance genes, we characterized the spread of the ability to become colistin-resistance via mobile plasmid. The IS*Kpn72*-containing mobile plasmid enables the strain to transit from colistin-sensitive to resistant phenotype by inactivating the *mgrB* gene, which may also explain the widespread of *mgrB* gene inactivation in clinical samples (Cannatelli et al., 2014; Gaibani et al., 2014). Interestingly, all copies of the IS*Kpn72* element contain a pair of 22 bp-length imperfect terminal inverted repeats (IR), which is a characteristic feature of the IS element in bacteria (Rezsöhazy et al., 1993; Mahillon and Chandler, 1998); but only the 12 copies of the IS*Kpn72* element located on the chromosome contain 9-bp direct repeated (DR) sequences, which are considered as a marker of IS insertion (Mahillon and Chandler, 1998). These observations indicate that the copies of IS*Kpn72* on the chromosome are probably inserted from the DR-missing IS*Kpn72* element on the pR10-341_2 plasmid. In addition, we observed a colistin-resistance acquirement of IS*chr* transformers (Figure 3C), which indicated that the bacteria also can store the ability to become colistin-resistance by inserting IS in chromosome. The ability to transit to colistin-resistant can be both stored at chromosome and disseminated through mobile plasmids.

The mechanism of a mobile plasmid-mediated transfer of the ability to transit from a colistin-sensitive to resistant phenotype brings not only challenges to colistin-resistance detection, but also raises concerns to the use of colistin in clinical treatment. Firstly, this type of colistin-resistant phenotype was generated by the insertion of an IS*Kpn72* element into the *mgrB* gene, which means the parental strain may be mis-classified as colistin-sensitive in drug-resistant genotyping or phenotyping assays. Secondly, the IS*Kpn72* and other IS elements widely exist in prokaryotes (Mahillon and Chandler, 1998; Frost et al., 2005), suggesting transition to colistin-resistance may be widely occurred (Cannatelli et al., 2013, 2014). Different methods have been deployed for antibiotic resistance analysis and prediction, including traditional antimicrobial susceptibility testing (AST) (Jorgensen and Ferraro, 1998) and bioinformatics tools (Zankari et al., 2012; Boolchandani et al., 2019). It is still a challenge to identify whether a strain is able to acquiring inheritable or transmissible antibiotic-resistance, or under specific conditions due to the wide existence of IS elements. Additionally, the frequency of the IS*Kpn72* insertion into the *mgrB* gene characterized in our study can reach ∼10^–6^, indicating that the IS insertion, especially the IS*4* family, should receive more attention in the clinical use of colistin.

## Data Availability Statement

The datasets presented in this study can be found in online repositories. The names of the repository/repositories and accession number(s) can be found in the article/Supplementary Material.

## Author Contributions

MN and YH conceptualized and designed the study. BZ and BY performed the experiments. BZ, WZ, XW, and SC analyzed the data. YW and ZS provided the materials. BZ, MN, and YH drafted the manuscript. BY and SC critically revised the manuscript. All authors read and approved the final manuscript.

## Conflict of Interest

The authors declare that the research was conducted in the absence of any commercial or financial relationships that could be construed as a potential conflict of interest.

## Footnotes

1 https://bigsdb.pasteur.fr/klebsiella/

2 https://clsi.org/standards/products/microbiology/documents/m100-preorder/

3 https://www.eucast.org/fileadmin/src/media/PDFs/EUCAST_files/General_documents/Recommendations_for_MIC_determination_of_colistin_March_2016.pdf

4 https://github.com/Albacore/albacore

5 http://www.genomicepidemiology.org/

6 http://www-is.biotoul.fr
