# Supplementary material for: Mobile Plasmid Mediated Transition From Colistin-Sensitive to Resistant Phenotype in Klebsiella pneumoniae
Source: Front Microbiol. 2021 Feb 15;12:619369. doi: 10.3389/fmicb.2021.619369 (PMC7917065; doi:10.3389/fmicb.2021.619369)
Supplement: Supplementary file 1 [file Table_1.docx]

**Supplementary Data**

**Mobile plasmid mediated transition from colistin-sensitive to resistant phenotype in *Klebsiella pneumoniae***

Baoyue Zhang, Bing Yu, Wei Zhou, Yue Wang, Ziyong Sun, Xiaojun Wu, Shiyun Chen, Ming Ni^*^, Yangbo Hu^*^

^*^Correspondence: Ming Ni [niming@tjh.tjmu.edu.cn](mailto:niming@tjh.tjmu.edu.cn) or Yangbo Hu [ybhu@wh.iov.cn](mailto:ybhu@wh.iov.cn)

**Supplementary Table 1.**  Primers used in this study.

| Name | Sequence (5’→3’) | Source |
| --- | --- | --- |
| mcr1-F1 | CGGTCAGTCCGTTTGTTC | (Yang et al., 2018) |
| mcr1-R1 | CTTGGTCGGTCTGTAGGG | (Yang et al., 2018) |
| mcr1-F2 | ATCAGCCAAACCTATCCCATCG | (Wang et al., 2018) |
| mcr1-R2 | ATAGGCATTGCTGTGCGTCTGC | (Wang et al., 2018) |
| mcr2-F1 | GCGATGGCGGTCTATCCTGTAT | (Wang et al., 2018) |
| mcr2-R1 | GCTGACACCCCACGTCATCGCA | this study |
| mcr2-F2 | TGTTGCTTGTGCCGATTGG | this study |
| mcr2-R2 | GTGTTACAGATGGTGTTGTTGGT | this study |
| mcr3-F1 | TTGGCACTGTATTTTGCATTT | (Yang et al., 2018) |
| mcr3-R1 | TTAACGAAATTGGCTGGAACA | (Yang et al., 2018) |
| mcr3-F2 | GATGGGTTACTATTGCTGG | this study |
| mcr3-R2 | CGATGAGCATCAGGGTAG | this study |
| mcr4-F1 | ATTGGGATAGTCGCCTTTTT | (Yang et al., 2018) |
| mcr4-R1 | TTACAGCCAGAATCATTATCA | (Yang et al., 2018) |
| mcr4-F2 | GTCATAGTGGTATAAAAGTACAG | (Wang et al., 2018) |
| mcr4-R2 | GTTGGCTCTGATAGACGGTGG | this study |
| mcr5-F1 | TATCTCGACAAGGCCATGCTG | (Yang et al., 2018) |
| mcr5-R1 | GAATCTGGCGTTCGTCGTAGT | (Yang et al., 2018) |
| mcr5-F2 | GCGGTTGTCTGCATTTATCAC | (Wang et al., 2018) |
| mcr5-R2 | TGCCGAAGACAGGTTTTCAAAG | this study |
| mcr6-F1 | GTCCGGTCAATCCCTATCTGT | (Wang et al., 2018) |
| mcr6-R1 | ATCACGGGATTGACATAGCTAC | (Wang et al., 2018) |
| mcr6-F2 | TAGGTAAGCTTGCCAGTATTGAG | this study |
| mcr6-R2 | CACCAACATACCGACATCACG | this study |
| mcr7-F1 | TGCTCAAGCCCTTCTTTTCGT | (Wang et al., 2018) |
| mcr7-R1 | TTCATCTGCGCCACCTCGT | (Wang et al., 2018) |
| mcr7-F2 | AGGGGATAAACCGACCCTGA | (Yang et al., 2018) |
| mcr7-R2 | TGATCTCGATGTTGGGCACC | (Yang et al., 2018) |
| mcr8-F1 | AACCGCCAGAGCACAGAATT | (Wang et al., 2018) |
| mcr8-R1 | TTCCCCCAGCGATTCTCCAT | (Wang et al., 2018) |
| mcr8-F2 | TACAATCGGCAACATAGCACT | this study |
| mcr8-R2 | CAACCACCAGTTTCGGTGGAG | this study |
| PhoPQ-F | CTGGGCGATTGCGCCAGCCTG | this study |
| PhoPQ-R | CACTGCAGGTGTCTGACAGGG | this study |
| PhoPR-Seq-F | CGCTGATGCTCCAGCTTTACCC | this study |
| PmrAB-F | CCGGGAGCAGTTGACCAATACCTATG | this study |
| PmrAB-R | CCTGATGAGGATAGCGCCCATGC | this study |
| PmrAB-seqF | GAAATCAGCGTCGGCAATCTGC | this study |
| mgrB-F | GGTATTGGCACTTAAGACCCAGAC | this study |
| mgrB-R | CCTCTTTCTCTGGCGCATTAG | this study |
| CrrAB-F | ATGCACAAGGTAAAGCCAGGTAG | this study |
| CrrAB-seqF | CTACTGCCGCGTCGATCTTATGC | this study |
| CrrAB-seqF | GTTGCAGAGTCTACACATTCACGG | this study |
| CrrAB-F2 | CTTATACATAAACATAAAGGTAGTCCGC | this study |
| mgrB-F | GGTATTGGCACTTAAGACCCAGAC | this study |
| mgrB-R | CCTCTTTCTCTGGCGCATTAG | this study |
| ISplas-F | CGGCTGTTGCGGGATCGAAC | this study |
| ISplas-R | CTCCCAATACGGTCAATCCGTGT | this study |
| ISchr-F | GAGCGCCTGATGCGGTATGCGCGGCCATCATCATCCTG | this study |
| ISchr-R | GATCTAAGCTTCTGCAGTGCGCAGCATATTCGCGATCG | this study |
| p15A-Sm-F | ACTGCAGAAGCTTAGATCCAGCGCTAGCGGAGTGTATAC | this study |
| P15A-Sm-R | TACCGCATCAGGCGCTCCGCTCAGTGGAACGAAAACTCAC | this study |
| Ec-F-F | CGGATTCACCACTCCAAGAATAATCACCCGAGTGTGATCATCT | this study |
| Ec-F-R | TATTCGCTGGTCACTTCGATGGTTTGC | this study |
| Kpn-F-F | TGCGCGGCCATCATCATCCTG | this study |
| Kpn-F-R | GCGCAGCATATTCGCGATCG | this study |
| P-F-F | CGGCTGTTGCGGGATCGAAC | this study |
| P-F-R | CTCCCAATACGGTCAATCCGTGT | this study |
